# Ultrasound guided corticosteroid injection for plantar fasciitis: a randomised controlled trial

**DOI:** 10.1186/1757-1146-4-S1-O29

**Published:** 2011-05-20

**Authors:** Andrew McMillan, Karl Landorf, Mark Gilheany, Adam Bird, Adam Morrow, Hylton Menz

**Affiliations:** 1 Department of Podiatry, La Trobe University, Melbourne, Victoria 3086, Australia; 2 Musculoskeletal Research Centre, La Trobe University, Melbourne, Victoria 3086, Australia

## Background

Plantar fasciitis is the most commonly reported cause of chronic pain beneath the heel. Management of this condition commonly involves the use of corticosteroid injection in cases where less invasive treatments have failed. However, despite widespread use, only two randomised trials have tested the effect of this treatment in comparison to placebo. These trials currently offer the best available evidence by which to guide clinical practice, though both were limited by methodological issues such as insufficient statistical power. Therefore, the aim of this randomised trial is to compare the effect of ultrasound-guided corticosteroid injection versus placebo for treatment of plantar fasciitis.

## Methods

The trial will be conducted at the La Trobe University Podiatry Clinic and will recruit 80 community-dwelling participants. Diagnostic ultrasound will be used to diagnose plantar fasciitis and participants will be required to meet a range of selection criteria. Participants will be randomly allocated to one of two treatment arms: (i) ultrasound-guided injection of the plantar fascia with 1 mL of 4 mg/mL dexamethasone sodium phosphate (experimental group), or (ii) ultrasound-guided injection of the plantar fascia with 1 mL normal saline (control group). Blinding will be applied to participants and the investigator performing procedures, measuring outcomes and analysing data. Primary outcomes will be pain measured by the Foot Health Status Questionnaire and plantar fascia thickness measured by ultrasound at 4, 8 and 12 weeks. All data analyses will be conducted on an intention-to-treat basis.

## Results

Data collection for the trial will be finalised in February 2011. Following this, statistical analyses of the trial data will be performed and the findings prepared for presentation.

## Conclusions

This will be a randomised trial investigating the effect of dexamethasone injection on pre-specified treatment outcomes in people with plantar fasciitis. Within the parameters of this protocol, the trial findings will be used to make evidence-based recommendations regarding the use of corticosteroid injection for treatment of this condition.

